# The effect of rollover footwear on head and trunk posture during standing

**DOI:** 10.1186/1757-1146-7-S1-A21

**Published:** 2014-04-08

**Authors:** Fateme Pol, Saeed Forghany, Christopher Nester, Atefe Rahimi

**Affiliations:** 1 Musculoskeletal Research Centre, Isfahan University of Medical Sciences, Iran; 2 Centre for Health Sciences Research, University of Salford, UK

## Background

Footwear with a curved sole profile has become popular due to the proposed benefits to gait, posture and altered muscle activity and tone. In addition, rocker profiled footwear are one of the most commonly prescribed therapeutic shoes [1-4]. The altered movement of the body over the foot due to the curved sole profile is assumed to alter the position of the hips and thereafter the trunk, spine and perhaps the head too. This could have some benefits for those with back pain [5]. However, whilst evidence for the effects on the lower limb is becoming comprehensive [1-4], there is a paucity of information for any effects on head and trunk posture.

## Aim

To investigate the effect of rollover footwear on head and trunk posture during standing.

## Material and methods

Head and trunk posture data of ten healthy female participants(age:24.5±1.8 years) was collected during one minute of quiet standing in two conditions (1) barefoot and (2) rollover footwear (Perfect Steps). The positions of 11 markers mounted on the spinous processes of S2, L5, L3, L1, T11, T9, T7, T5, T3, C7 and midpoint of forehead were collected by an eight-camera motion capture system at 100 Hz. The planar angles of head and neck, and the radii of thoracic and lumbar curve in the sagittal plane were calculated using an approach described by Forghany et al [6].

## Results

The radius of lumbar curve was decreased significantly by 11.2% wearing the rollover footwear compared to the barefoot condition (p<0.05) (i.e. spine more extended). The radius of thoracic curve also increased (i.e. spine also more extended) but the differences were not statistically significant (p=0.3). Participants wearing the rollover footwear showed significant increase in the planar angles of head and neck by 7.8% in comparison with barefoot condition (p = 0.01) (i.e. head and neck more extended). (Table 1)

**Table 1:** Head and trunk postural changes wearing the rollover footwear in comparison with barefoot condition during standing.

	Barefoot	Rollover footwear	P value	%differences
Radius of lumbar curve (cm)	16.6	14.8	0.0003	-10.8
Radius of thoracic curve (cm)	39.6	41.5	0.3	4.8
Head-C_7_ angle (degree)	34.8	37.5	0.01	7.8

## Conclusion

The current study showed that rollover footwear is able to modify head and trunk posture in quiet standing compared to barefoot. Participants stood more erect in footwear than barefoot. These shoes could therefore have a role in the management of some conditions where an increase in back extension is desirable but the clinical relevance of those changes remains to be determined.
